# Heavy metals and metalloid distribution in different organs and health risk assessment for edible tissues of fish captured from Honghu Lake

**DOI:** 10.18632/oncotarget.21901

**Published:** 2017-10-13

**Authors:** Jingdong Zhang, Liyun Zhu, Fei Li, Chaoyang Liu, Zhaofei Yang, Zhenzhen Qiu, Minsi Xiao

**Affiliations:** ^1^ Research Center for Environment and Health, Zhongnan University of Economics and Law, Wuhan 430073, China; ^2^ School of Information and Safety Engineering, Zhongnan University of Economics and Law, Wuhan 430073, China

**Keywords:** toxic metals, Honghu Lake, fish consumption, carcinogenic risk, estimated weekly intake

## Abstract

Honghu Lake is the seventh largest freshwater lake in China, and fishery is one of the most important economic sources for local inhabitants. Toxic metal concentrations in muscle of all analyzed fish species captured from Honghu Lake were generally below China standards, except Cr in crucian carp. The average concentrations were decreased in the following order, Zn (14.65 mg/kg) > Cr (1.25 mg/kg) > Cu (0.5994 mg/kg) > Pb (0.0884 mg/kg) > Cd (0.0069 mg/kg) > As (0.0007 mg/kg). There was no significant health risk in consuming fish captured from Honghu Lake, based on the analysis results of target hazard quotient (THQ), carcinogenic risk (CR), and estimated weekly intake (EWI). Mixed edible fish tissues consuming brought higher carcinogenic risks than muscle consuming. Pb was regarded as the major contributor of potential non-carcinogenic risk, while As of the potential carcinogenic risk. THQ set the most stringent allowed values of the average consuming amount of fish muscle at 1,316 g/d, while CR set the value of mixed fish tissues at 525 g/d.

## INTRODUCTION

With the rapid development of industry and agriculture, heavy metal and metalloid pollution aroused highly concern for its features like toxicity, persistence, and bioaccumulation, particular in some developing countries like China, Pakistan and India [1–8]. Heavy metals and metalloids were hard to degrade, and could easily transfer and continuously accumulate in water, sediment, aquatic organisms, and finally accumulate in human body through drinking, touching, and ingestion [9, 10]. Fish, an important part for human diet, was a reliable bioindicator for environmental metals accumulation monitoring, because fish occupied a relatively high trophic level [11–14]. Toxic metals in edible fish tissues, which can even cause acute or chronic poisoning, can directly do harm to human health [15–17]. For example, excessive Pb ingestion may cause neurotoxicity, nephrotoxicity, and many other adverse health effects [18, 19]. Some essential elements, like Cu which is essential for metabolic function, can also cause health problems with symptoms like nausea, vomiting, lethargy and fatigue when their concentrations beyond the corresponding recommended dietary allowances [20]. Furthermore, Cu may cause an additive toxic effect in association with Zn and Hg [21].

Honghu Lake, the seventh largest freshwater lake in China, was located in the middle of Hubei province, between the Yangtze River and Dongjing River. Previous researches revealed that the surface water in Honghu Lake has been gradually polluted by heavy metals and metalloids due to anthropogenic activities [22–25], and Cr was recommended as the primary pollutant both in sediment and surface water [26]. Fishery was one of the most important sources for local inhabitant diet and local economy [27]. The output value of fishery in Honghu area arrived at 7, 249 million rmb, occupied 57% of the total output value of agriculture (12, 712 million rmb), in 2016 [28]. Previous researches in health risk assessment for heavy metals and metalloid through fish consumption mostly focus on fish muscle consumption [29–31]. However, some other edible fish tissues, like bladder, skin, liver, that contained abundant nutrients were also recognized as valuable food ingredients and were popular with local inhabitants [32–34]. For example, sturgeon bladder contained substantial bone collagen which can promote human growth hormone secretion, maintain bone toughness and even may delay aging [33]. Besides, some researches also revealed that active fish tissues, particular like liver and gill, had higher heavy metal accumulated abilities than fish muscle [35–37]. Thus, it is necessary to investigate the heavy metal and metalloid distribution in different tissues of fish captured from Honghu Lake, and to compare the corresponding health risks brought by fish muscle consumption and mixed fish tissues consumption.

The specific objectives of this study are as follows: (1) to investigate the As, Cd, Cr, Cu, Pb, and Zn concentration distributions in muscle and other tissues of different kinds of fish captured from Honghu Lake; (2) to assess the human health risks by fish muscle and mixed fish tissues consumption based on the target hazard quotient (THQ) assessment method, the carcinogenic risk (CR) assessment method and the estimated weekly intake (EWI) assessment method; (3) to analyze the correlation between the detected trace elements in fish muscles and between different organs to specific element.

## RESULTS AND DISCUSSION

### Heavy metals and metalloid concentration distribution

#### Heavy metals and metalloid distribution in surface water

Surface water samples were collected from 20 sampling sites throughout Honghu Lake. The mean heavy metals and metalloid concentration in surface water decreased in the order of Zn (18.04 μg/L) > Pb (3.42 μg/L) > Cu (3.09 μg/L) > Cr (1.63 μg/L) > As (0.99 μg/L) > Cd (0.14 μg/L). The concentrations of all the selected trace elements were within the GradeⅠof surface water standard and the permissible limits of drink water guidelines of WHO and USEPA.

### Heavy metals and metalloid distribution in fish muscle

The concentrations of detected trace elements (As, Cd, Cr, Cu, Pb and Zn) in muscle of 6 kinds of fish captured from Honghu Lake were listed in Table 1.

**Table 1:** Trace elements concentrations in muscle of fish captured from Honghu Lake

Fish species	Heavy metals and metalloid contents (Mean + SD) (mg/kg-wet wt.)					
	As	Cd	Cr	Cu	Pb	Zn
Bighead carp	0.0000	0.0099	0.6633	0.1061	0.0835	9.25
	± 0.0000	± 0.0019	± 0.0920	± 0.0773	± 0.0100	± 0.73
Crucian carp	0.0000	0.0087	3.36	0.3829	0.0938	15.31
	± 0.0000	± 0.0049	± 0.0036	± 0.0786	± 0.0004	± 1.46
Grass carp	0.0000	0.0032	0.5369	0.4502	0.0111	17.29
	± 0.0000	± 0.0002	± 0.4395	± 0.0726	± 0.0111	± 3.02
Mandarin fish	0.0000	0.0066	1.70	0.2398	0.0639	10.98
	± 0.0000	± 0.0004	± 0.4952	± 0.1716	± 0.0232	± 5.12
Small crucian carp	0.0000	0.0072	0.8760	0.5171	0.1536	19.30
	± 0.0000	± 0.0032	± 0.5790	± 0.5157	± 0.2661	± 2.78
Yellow catfish	0.0040	0.0056	0.3680	1.90	0.1242	15.77
	± 0.0042	± 0.0060	± 0.1621	± 1.27	± 0.0622	± 4.15
**Range**	NA−0.01	NA−0.02	NA−3.36	0.03−3.20	NA−0.61	5.86−22.54
Mean	0.0007	0.0069	1.25	0.5994	0.0884	14.65
Standard	0.1	0.1	2	50	0.5	50

^*^ NA: Not available

Arsenic (As) could only be detected in yellow catfish muscle with the low average concentration of 0.004 mg/kg, about 1/25 of 0.1 mg/kg (the corresponding limitation value) [38].

Cadmium (Cd) concentrations in fish muscle stayed at a relatively stable value range in various fish kinds. The highest level of Cd was in bighead carp muscle, and the lowest was in grass carp muscle, in comparison. Cd concentrations of all the fish muscles were lower than 0.01 mg/kg, approximately 1/10 of the corresponding limitation value (0.1 mg/kg) [38].

Chromium (Cr) concentrations in fish muscle were absolutely different in different kinds of fish. Cr concentrations were much higher in crucian carp muscle and mandarin fish muscle than the others. In addition, Cr concentration in crucian carp muscle is even over the safety limitation, 2.0 mg/kg [38].

Lead (Pb) concentration levels didn't show significant differences in various fish. With the highest level of Pb in small crucian carp, the lowest lied in the grass carp. The average concentration was 0.088 mg/kg, and Pb concentrations were increased in the following order: grass carp < mandarin fish < bighead carp < crucian carp < yellow catfish < small crucian carp. They were all lower than their corresponding limitation values, 0.5 mg/kg [38].

Copper (Cu) concentration levels were ranged from 0.11–1.90 mg/kg, and its average concentration was 0.60 mg/kg. Zinc (Zn) concentration levels were ranged from 9.25–19.30 mg/kg, with the average level of 14.65 mg/kg. They were all much lower than their corresponding limitation values, 50 mg/kg [39].

Toxic metals concentrations in muscle of all kinds of fish were significant lower than the China's standards except Cr, and their average concentrations were increased in the following order: As < Cd < Pb < Cu < Cr < Zn. Bighead fish had the highest Cd concentration level and the lowest Cu and Zn levels. Yellow catfish had the lowest Cr concentration level and the highest As and Cu levels. Crucian carp had the highest Cr concentration level. Small crucian carp had the highest Pb and Zn concentration levels compares to grass carp has the lowest Cd and Pb levels. Thus, for local residents, grass carp was recommended as the daily fish consuming species, which had the relatively lower toxic metals concentrations, and followed by yellow catfish and mandarin fish.

Compared to the other lakes and rivers in China (Table 2), the average Cr concentration in muscle of fish captured in Honghu Lake was relatively higher while the As concentration was relatively lower. Compared to the other lakes and rivers in Asia, almost all detected trace elements in muscle of fish captured in Honghu Lake were relatively lower than in the other countries’ lakes or rivers. However, the trace elements bioaccumulation rank and order of magnitude in muscle of fish captured in Honghu Lake were roughly coincident with fish captured in the other lakes or rivers [12, 14, 37, 40–48].

**Table 2:** Trace element concentrations in fish muscle reported from literature

Sample area	Heavy metals and metalloid concentration (Mean) (mg/kg)						Nation	References
	As	Cd	Cr	Cu	Pb	Zn		
Poyang Lake	0.0040	0.0042	0.2380	0.3670	0.0315	7.23	China	[37]
Taihu Lake	-	0.012	0.945	-	0.232	73	China	[40]
Pearl River Delta	0.78	0.04	0.43	1.53	0.78	22.4	China	[12]
Yangtze River	0.019	0.100	0.244	1.70	0.525	7.07	China	[14]
Bangshi River	3.55	0.30	1.12	22.80	4.64	169.0	Bangladesh	[41]
Kolleru Lake	-	0.10	0.19	0.31	0.12	0.44	India	[35]
Hooghly River	-	0.91	1.95	32.10	16.18	28.44	India	[42]
Kichera River	-	0.05	-	-	0.19	4.37	Russia	[43]
Firat (Euphrates) River	0.0737	0.0005	0.5270	0.3850	0.0530	3.39	Turkey	[44]
Chenab River	1.12	0.10	0.21	4.03	0.14	36.5	Pakistan	[45]
Cempaka Lake	-	0.01	1.50	0.85	0.04	11.25	Malayia	[46]
Southern U.S waterbody	0.053	-	0.313	0.360	0.724	-	Southern U.S	[47]
Iran waterbody	0.243	0.025	0.147	5.67	0.288	5.45	Iran	[48]
Honghu Lake	0.0007	0.0069	1.25	0.5994	0.0884	14.65	China	This study

The calculation results of the Bio-concentration factor (BCF) in muscle of fish captured from Honghu Lake are listed in Table 3. BCF value more than 1 means aquatic organism probably has accumulated trace elements from aquatic environment but is not significant unless the BCF value exceed 100 [44, 49]. Cr and Zn had significantly higher accumulation in each study fish species captured from Honghu Lake. Cu showed a relatively high accumulation from Honghu Lake surface water in yellow catfish, and its accumulation also obvious in crucian carp, grass carp and small crucian carp. The diverse BCF values revealed the different ability for different fish species absorb different trace elements from water. Crucian carp showed obviously high accumulation ability in absorbing Cr, while yellow catfish in Cu and small crucian carp in Zn.

**Table 3:** The Bio-concentration factors (BCFs) in different organs of different kinds of fish captured from Honghu Lake

	As	Cd	Cr	Cu	Pb	Zn
Mean content in water (μg /L)	0.990	0.140	1.630	3.090	3.420	18.04
BCF in muscle of different fish						
Bighead carp	0	71	407	34	24	513
Crucian carp	0	62	2059	124	27	848
Grass carp	0	23	329	146	3	958
Mandarin fish	0	47	1041	78	19	609
Small crucian carp	0	51	537	167	45	1070
Yellow catfish	4	40	226	615	36	874
BCF in different fish organs						
Bladder	28.46	590.5	284.2	182.4	113.5	1868
Gill	2.93	19.75	304.7	396.7	84.5	3810
Intestine	7.62	224.7	127.2	901.1	29.77	4057
Liver	1.68	180.0	190.0	904.0	145.6	2982
Muscle	0.00	50.9	959.1	95.4	18.45	732
Scale	0.05	3.62	364.8	212.2	194.2	39332
Skin	0.65	46.22	68.5	891.8	9.34	1407

### Heavy metals and metalloid distribution in different fish organs

**Figure 1:**
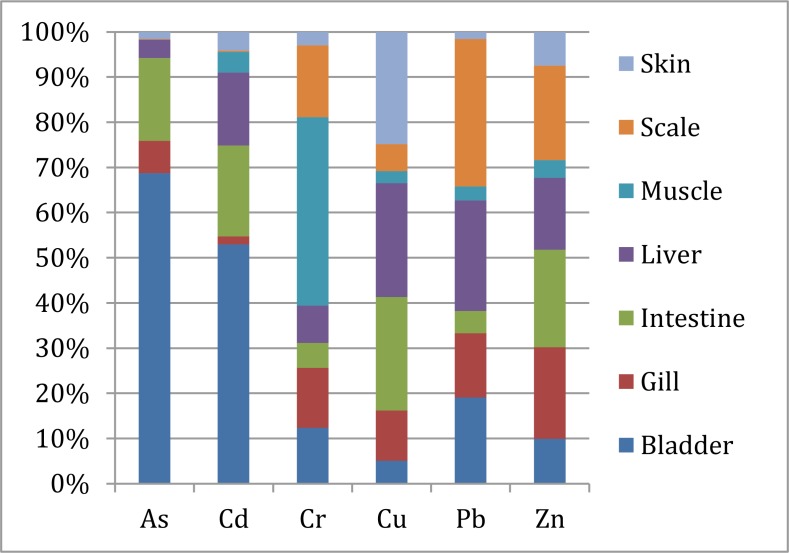
Trace elements concentration proportion and distribution in different fish organs from Honghu Lake

Figure 1 shows the different detected trace elements proportion and distribution in different kinds of fish organs.

The concentration of Zn was higher than the others in each fish organ, while the concentrations of Cr and Cu were slightly higher. The concentrations of As and Cd were higher in fish bladder (0.028 mg/kg and 0.083 mg/kg) than the other parts of fish, while in fish intestine were slightly higher. The highest concentration of Cr lied in fish muscle (1.56 mg/kg), followed by scale (0.595 mg/kg), gill (0.497 mg/kg) and bladder (0.463 mg/kg). The Cu concentrations in fish liver (2.79 mg/kg), intestine (2.78 mg/kg) and skin (2.76 mg/kg) were 3–5 times higher than the other organs. The Pb concentrations decreased in the following order, scale (0.664 mg/kg) > liver (0.498 mg/kg) > bladder (0.388 mg/kg) > gill (0.289 mg/kg) > intestine (0.102 mg/kg) > muscle (0.063 mg/kg) > skin (0.032 mg/kg). The Zn concentrations didn't show a considerable distinction in its distribution of most organs, and in fish muscle (13.21 mg/kg) the concentration was slightly lower than the other parts.

The calculation results of the Bio-concentration factor (BCF) in different organs of fish captured from Honghu Lake are listed in Table 3. Cr, Cu and Zn had significantly higher accumulation in each parts of fish captured from Honghu Lake. Cd significantly accumulated in fish bladder, intestine and liver, While Pb was significantly accumulated in fish bladder, liver and scale. Fish bladder showed obviously high accumulation ability in absorbing As and Cd, while muscle in Cr, liver in Cu, scale in Pb and intestine in Zn.

**Figure 2:**
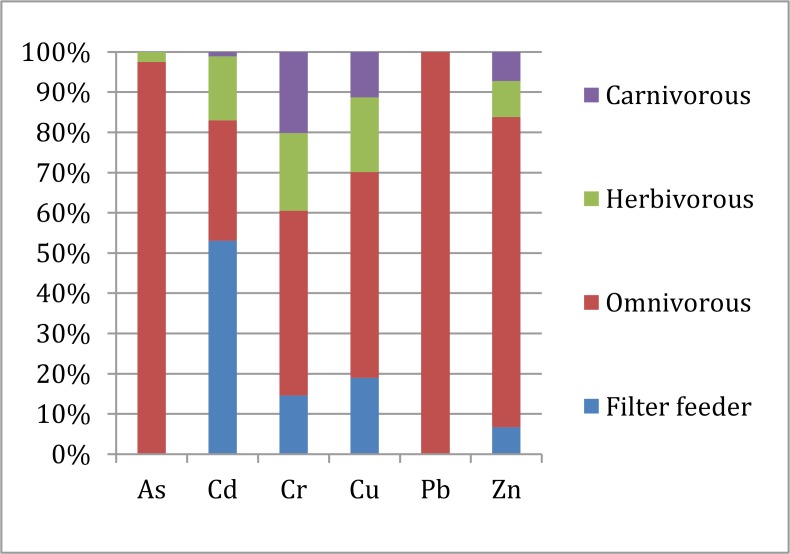
Trace elements concentration proportion in intestine of fish with different feeding habits

Overall, trace elements concentrations, except the Cr concentration, in muscle were relatively lower than the other tissues of fish captured from Honghu Lake. The different toxic metals distributions in fish organs may due to their different absorption and metabolism abilities for each element. Muscle is not an active tissue for most heavy metal bioaccumulation. Gill, scale and skin are organs directly exposed in the aquatic environment. The trace elements concentrations in fish gill, scale and skin probably have direct relationships with the trace element concentration in lake water. However, the trace elements concentrations detected in fish gill, scale and skin were relatively lower than the other parts. Trace elements concentrations in fish intestine possibly root in ingestion and probably influenced by different feeding habits. Figure 2 proves that carnivorous has the obviously high accumulation of each trace element in its intestine. Liver is the principle metabolism function organ in fish, and can easily storage heavy metals and metalloid from aquatic environment and ingestion. Most trace elements were mainly accumulated in fish bladder, liver and intestine. Researches which haven't taken fish bladder into consideration, mainly found toxic metals accumulated in liver [13, 36] and gill [36, 50]. With the expansion of the study objects scope, some researchers found other tissues, like bladder [37] and intestine [51], also had high toxic metals accumulation abilities, and showed a roughly consistency with our investigation results.

### Heavy metals and metalloid distribution difference between in fish muscle and in mixed fish tissues

**Figure 3:**
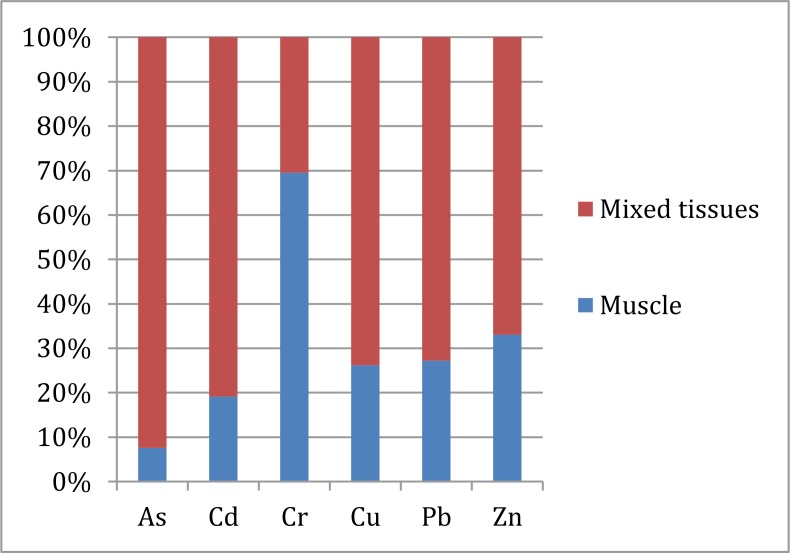
Trace elements concentration proportion in muscle and mixed tissues of fish

Toxic metals concentration distribution shows a significantly difference in fish muscle and mixed edible fish tissues (liver, skin, bladder and muscle). Figure 3 illustrates that all the detected trace elements concentration in mixed fish tissues were significantly higher than in fish muscle, except Cr.

**Figure 4:**
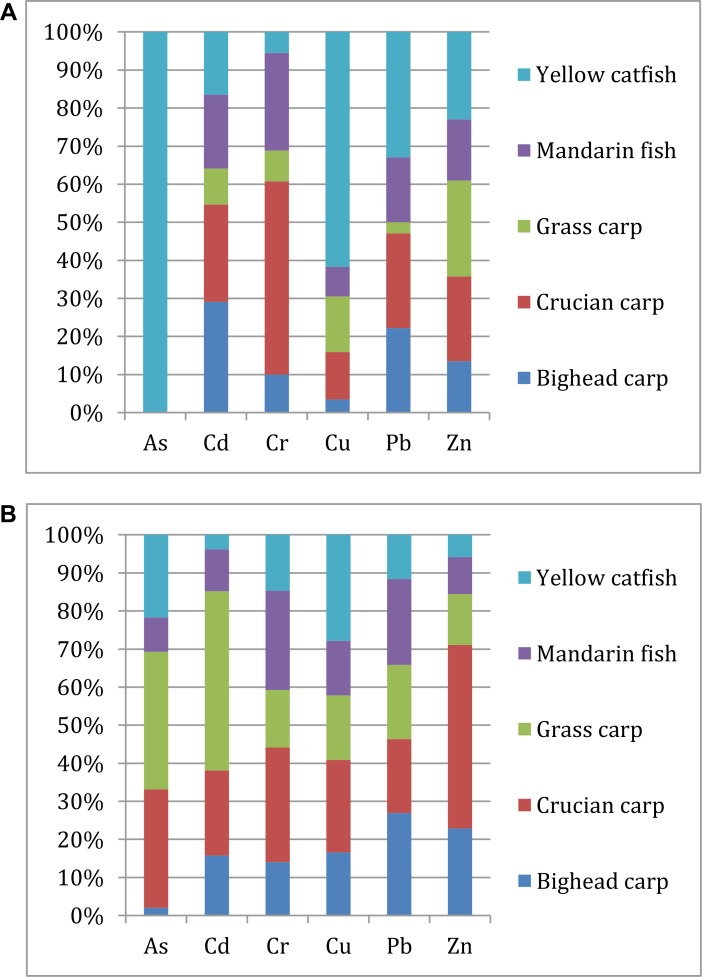
Trace elements concentration proportion and distribution in muscle (**A**) and mixed tissues (**B**) of different kinds of fish.

Figure 4A shows the mean trace elements concentration distribution in fish muscle of different kinds of fish. The As in fish muscle could only be detected in yellow catfish (0.004 mg/kg), and the highest Cu and Pb concentration levels were in yellow catfish (1.90 mg/kg and 0.124 mg/kg). The mean Cd concentrations of fish muscle decreased in the order of bighead carp (0.010 mg/kg) > crucian carp (0.009 mg/kg) > mandarin fish (0.007 mg/kg) > yellow catfish (0.006 mg/kg) > grass carp (0.003 mg/kg). The Cr concentration of crucian carp (3.36 mg/kg) was significantly higher than the other kinds of fish, while the grass carp (0.368 mg/kg) was considerable lower than the other kinds of fish. No obvious difference was found in the Zn concentration distribution in different kinds of fish, except the Zn concentration in bighead carp (9.25 mg/kg) and mandarin fish (10.99 mg/kg) were slightly lower than the other kinds of fish.

Figure 4B shows the mean heavy metal concentration distribution in mixed tissues of different kinds of fish. The As could be detected in each fish and its concentration reach the highest point in the grass carp (0.011 mg/kg). The Cu concentration in yellow catfish (2.44 mg/kg) still lied the highest stage, while the Pb concentration in yellow catfish (0.153 mg/kg) fell to the rock bottom. On the contrary, the Cd concentration in grass carp (0.044 mg/kg) rushed to the highest point (it was the bottom when only take muscle into consideration). The Cr concentration in crucian carp (0.757 mg/kg) was still significantly higher than the other, while the Zn concentration in crucian carp (99.1 mg/kg) also got the highest point, too.

In conclusion, yellow catfish and crucian carp had a relatively higher toxic metals accumulation in fish muscle, and crucian carp and grass carp had a relatively higher accumulation in mixed fish tissues.

### Health risk assessment in edible tissues of different fish

#### Calculation results of the target hazard quotient (THQ)

Table 4 shows the selected R_FD_ values [52] of 6 heavy metals and the calculated results of the target hazard quotient for fish consumption [37]. The average individual fish muscle consumption in Hubei is 54.33 g/d [53]. The calculated results of THQ were based on the average trace elements concentration in fish muscle and mixed tissues.

**Table 4:** Calculation results of target hazard quotients (THQ), carcinogenic risk (CR) and estimated weekly intake (EWI) from fish muscle and mixed tissues consumption

Elements	Mean Concentration (mg/kg)		R_FD_ (mg/kg/d)	Target hazard quotients (THQ)		Carcinogenic risk (CR)		Estimated weekly intake (EWI) (μg/kg)		PTWI^a^ (μg/kg)	EWI/PTWI	
	Muscle	Mixed tissues		Muscle	Mixed tissues	Muscle	Mixed tissues	Muscle	Mixed tissues		Muscle	Mixed tissues
As	0.0007	0.0082	0.0003	0.0020	0.0230	8.88E-07	1.03E-05	0.0043	0.0503	15	0.03%	0.34%
Cd	0.0069	0.0292	0.0010	0.0058	0.0247			0.0426	0.1800	7	0.61%	2.57%
Cr	1.2496	0.5474	1.5000	0.0007	0.0003			7.7149	3.3794	15	51.43%	22.53%
Cu	0.5994	1.6874	0.0400	0.0127	0.0357			3.7006	10.4175	3500	0.11%	0.30%
Pb	0.0884	0.2358	0.0040	0.0187	0.0499	6.35E-07	1.70E-06	0.5458	1.4558	25	2.18%	5.82%
Zn	14.6499	29.6003	0.3000	0.0413	0.0834			90.4465	182.7484	7000	1.29%	2.61%

^a^PTWI: provisional tolerable weekly intake (JECFA, 1993).

The THQ values of each trace metal based on the average concentrations in fish muscle were decreased in the following order, Zn > Pb > Cu > Cd > As > Cr. Without considering the nutrient elements (Zn and Cu), Pb (accounts for nearly 50% of the total target hazard quotient) was the major contributor of non-carcinogenic risk to the inhabitant in fish muscle. This conclusion shows a consistency with the results in previous studies [14, 44, 54].

Edible parts of fish for Hubei inhabitants not only include its muscle, but also include most of its organs like skin, bladder and liver [32–34]. Chaffy dish with mixed fish tissues is a very common and popular daily diet for Honghu residents. Table 4 compares the calculated results of the target hazard quotient for the consumption of fish muscles and mixed fish tissues (including fish muscle, liver, bladder and skin). The calculated results of THQ were based on the average heavy metal concentration of wild fish muscle and mixed wild fish tissues. All the THQ values of As, Cd, Cr, Cu, Pb and Zn were less than 1, and that means there are no significant health risk in this elements from fish muscle and mixed tissues consumption. Table 4 also shows that the THQ values of most trace metals in mixed fish tissues were several times higher than the THQ values in fish muscle, while the Cr THQ value in mixed fish tissues were slight lower than in muscle. The THQ values of mixed wild fish tissues followed in the descending order, Zn > Pb > Cu > Cd > As > Cr. Without considering the nutrient elements (Zn and Cu), Pb also was the major contributor of non-carcinogenic risk to the inhabitant in mixed fish tissues. The limitations of fish consumption based on THQ are listed in Table 5. Local people were allowed consuming less than 1, 316 g wild fish muscle or 651 g mixed wild fish tissues for an individual a day to assure their health.

**Table 5:** Calculation results of food consumption rate (F_IR_) limitation based on target hazard quotients (THQ), carcinogenic risk (CR) and estimated weekly intake (EWI)

F_IR_ limitation (g/d)	As	Cd	Cr	Cu	Pb	Zn
Based on target hazard quotients (THQ)						
Muscle	27531	9310	77112	4287	2907	1316
Mixed tissues	2365	2204	176040	1523	1090	651
Based on carcinogenic risk (CR)						
Muscle	6118				8549	
Mixed tissues	525				3205	
Based on estimated weekly intake (EWI)						
Muscle	9240000	437449	5176	2517851	121946	206036
Mixed tissues	793620	103547	11816	894420	45717	101972

### Calculation results of the carcinogenic risk (CR)

The carcinogenic risk calculation results of As and Pb for fish consumption are listed in Table 4. Acceptable carcinogens risk level range was from 10^–4^ to 10^–6^, and carcinogens risk lower than 10^–6^ was recognized as negligible compares to higher than 10^–4^ was recognized as unacceptable. Both of the As and Pb carcinogenic risks in fish muscle were negligible, and the As and Pb carcinogenic risks in mixed fish tissues were within the acceptable range of 10^–4^ to 10^–6^. Therefore, there were no significant health risks in local inhabitant consuming fish captured from Honghu Lake, and consuming mixed tissues of fish would bring higher carcinogens risks than consuming fish muscle separately.

The limitations of fish muscle and mixed tissues consumption based on CR are listed in Table 5. Local people were allowed consuming less than 6,118 g wild fish muscle or 525 g mixed wild fish tissues for an individual a day to assure their health.

### Calculation results of the estimated weekly intake (EWI)

The estimated weekly intakes of trace elements from fish muscle and mixed tissues consumption to the local residents are listed in Table 4. Each EWI value of trace metals was significantly lower than the respective provisional tolerable weekly intakes (PTWIs). In other words, there was no significant risk in consuming fish captured from Honghu Lake, when only took As, Cd, Cr, Cu, Pb and Zn into consideration. EWI/PTWI values were selected to compare the potential health risks of different trace metals equitably. The EWI/PTWI values of wild fish muscle were decreased in the following order, Cr > Pb > Zn > Cd > Cu > As. The EWI/PTWI values of mixed wild fish tissues were in the descending order, Cr > Pb > Zn > Cd > As > Cu. The above calculation results reveal that Cr and Pb were the major contributors of non-carcinogenic risk to the local inhabitants for fish consuming. This conclusion shows a consistency with the results in previous study [29]. For fish captured from Honghu Lake, As has a significantly higher bioaccumulation in mixed fish tissues, and mixed fish tissues could bring more potential health risks than muscle in each element except Cr.

The limitations of fish consumption based on EWI are listed in Table 5. Local people were allowed consuming less than 5,176 g wild fish muscle or 11,816 g mixed wild fish tissues for an individual a day to assure their health. In conclusion, THQ set the allowed values of the food consumption rate at 1,316 g/d wild fish muscle or 651 g/d mixed wild fish tissues for an individual, while this values in CR were 6,118 g/d wild fish muscle or 525 g/d mixed wild fish tissues, and in EWI this values were 5,176 g/d wild fish muscle or 11,816 g/d mixed wild fish tissues. Thus, for local residents, consuming less than 1,316 g/d wild fish muscle or less than 525 g/d mixed wild fish tissues can assure their health to the most extent.

### Heavy metals and metalloid correlation analysis

#### The Pearson correlation analysis between trace elements in fish muscle

Correlation matrix can reveal the viable relations among heavy metals, which can provide essential information for source apportionment and pollution pathway analysis. Pearson's correlation analysis required each variable presents normal distribution, and thus Kolmogorov-Smirnov (K-S) test was employed in testing the collected data before correlation analysis. The Cd, Cr, Pb and Zn concentration values were normal distributed (*P* > 0.05), while the As and Cu concentration values were not accordant (*P* < 0.05) and needed to be conversed through its orders. The relationships between the trace elements in muscle of fish captured from Honghu Lake were analyzed by Pearson's correlation analysis, and the calculation results were demonstrated in Supplementary Table 1. Positive relationship in significance (*P* < 0.05) was observed between Cu and Zn (*r* = 0.834) in fish muscle, while the other relationships between each two elements were not so significant. The positive significant relationships between elements could not always be deduced to a common source, however, it would truly give out some useful support information in finding the pollution sources or enrichment pathways for toxic metals.

### The Pearson correlation analysis between fish organs for trace elements

The relationships between different fish organs for different trace elements were analyzed by Pearson's correlation analysis, and the calculation results were demonstrated in Supplementary Table 2. For As, positive relationship in significance (*P* < 0.01) was found between intestine and scale (*r* = 0.882), and positive relationship in significance (*P* < 0.05) was found between intestine and gill (*r* = 0.783). For Cr, positive relationship in significance (p < 0.05) was found between muscle and gill (*r* = 0.761). For Cu, positive relationship in significance (*P* < 0.01) was found between bladder and gill (*r* = 0.877). For Pb, negative relationship in significance (*P* < 0.01) was found between skin and liver (*r* = −0.868). For Zn, positive relationship in significance (*P* < 0.01) was found between intestine and liver (*r* = 0.889), and positive relationship in significance (*P* < 0.05) was found between intestine and muscle (*r* = 0.709).

Significant positive relationships between fish intestine and the other organs found in absorbing As and Zn probably reveals that ingestion was the main way for trace elements accumulation in fish. Similarly, significant positive relationship between fish gill and the other organs in absorbing As, Cr and Cu reveals that gill was considered as the central organs for fish absorbing heavy metals and metalloid from its aquatic environment. Contrarily, significant negative relationship between fish skin and the other organs probably reveals the limited capacity of fish to absorb heavy metals from water through dermal contact.

## MATERIALS AND METHODS

### Study area

Honghu Lake is located in the middle of Hubei province, and has 2 channels to exchange water with the Yangtze River. Honghu Lake is the seventh largest freshwater lake in China and the largest nature wetland reserved in Hubei province. The surface size of Honghu Lake is about 348.3 km^2^ with 23.4 km in the length from east to west and 20.8 km in the width from north to south. The average depth of this lake is nearly 1.35 m, with the maximum water depth of 2.3 m and the minimum water depth of 0.4 m [55]. The climate of Honghu Lake belongs to subtropical monsoon, and the average precipitation around this lake is 1,061–1,331 mm and the average air temperature is 19 °C. Honghu city is rich in its fish resources, and its fish yield is in the second place of all china's counties and cities. Honghu Lake had 182 kinds of fish before 50s, 114 kinds of fish in 60s, and 89 kinds of fish in 70s, and this number continuously dropped to 59 in 2013. All the fish species belong to 7 orders and 18 families, and carp accounts for about 58.5%. Ferocious carnivorous fish account for 57.4%, omnivorous fish account for 22.2%, herbivorous fish account for only 7.4%, and filter fish account for 13% [56]. Excessive discharge of domestic sewage, agricultural non-point source pollution, excessive fishery and aquaculture obviously degrade the water quality and the carrying capacity of the Honghu Lake ecosystem [26].

### Sample collection and preparation

Water samples were collected at 0.5 m below the lake surface from 20 sampling sites, Supplementary Figure 1 which were selected based on the method of mesh point sampling and current national hydrologic conditions. All the water samples were filtered through 0.45 μm millipore filters, and then were collected into 1 L pre-conditioned acid-washed polyethylene containers. All the samples were transported to laboratory within 24 hours, and were cryopreserved in the temperature of −4°C [23].

Fresh fish samples were collected from Honghu Lake helped by local fishermen. To catch more kinds of fish, fisherman set the gill nettings in the surface and middle of the lake and fish cages in bottom. The essential information of fish capture from Honghu Lake was listed in Table 6. All of the fish samples were carefully preserved in polyethylene sealing bags, and both of its length and weight were roughly recorded. All the samples were cryopreserved in the temperature of −20°C and transport to laboratory as soon as possible. All the fish samples were carefully cleaned by the ultra pure water and disassembled into 7 parts (bladder, gill, intestine, liver, muscle, scale and skin) respectively by stainless steel tools. Each part was gently dried by disposable filter paper and homogenized by meet masher. All of the homogenized parts of fish samples were carefully preserved in small polyethylene bottle with category labels and transferred into refrigerator in the temperature of −20°C.

**Table 6:** Essential information of fish captured from Honghu Lake

Common name	Scientific name	Feeding habits	*n*	Length/cm	Weight/g
Bighead carp	Hypophthalmichthys nobilis	Filter feeder	8	37.1–41.8	751–796
Crucian carp	Carassius auratus	Omnivorous	8	18.4–22.6	161–189
Grass carp	Ctenopharyngodon idellus	Herbivorous	8	32.2–38.9	550–601
Mandarin fish	Siniperca chuatsi	Carnivorous	8	29.3–33.7	525–570
Small crucian carp	Ctenopharyngodon idellus	Omnivorous	16	8.2–12.6	89–110
Yellow catfish	Pelteobagrus fulvidraco	Carnivorous	24	19.3–23.7	59–82

All regents used, including nitric acid (65%) and hydrogen peroxide (30%), were of ultra-pure grade (Shanghai Sinopharm Group Chemical Reagent Limited Company). All experiment vessels for sample storage, digestion and detection were immersed overnight in nitric acid (20–30%) solution, rinsed in ultrapure water and dried in clean laboratory oven.

### Sample digestion and analysis

For water samples, the digestion methods were referred to “Water quality- digestion of total metals-nitric acid digestion method (HJ 677–2013)” [57] and “Water quality-determination of mercury, arsenic, selenium, bismuth and Antimony-Atomic Fluorescence Spectrometry (HJ 694–2014)” [58]. For fish samples, the digestion procedures were carefully followed the individual trace element detection methods from National food safety standards [59–64]. Approximately 0.5 g fish tissues were weighted into the digestion vessel with 8 ml nitric acid and 2 ml hydrogen peroxide. Vessels with mixed solution were closed and stand 20–30 minutes in the room temperature, then transferred to the microwave digestion system with the designed heated programming. After digestion, all solutions were replaced into small porcelain mugs on electric platen by base solution (0.2% diluent nitric acid), heated at 120 °C until only 2–3 ml digestion solutions left, to drive away the residual acid. Then, the left solutions were diluted into 10 ml colorimetric tubes for storage and further detection by the base solution (0.2% diluent nitric acid). Cu, Zn, Cr, Cd, Pb were detected with Atomic Absorption Spectroscopy (AAS) and As was detected by Atomic Fluorescence Spectrometry (AFS) under appropriate analytical conditions.

Quality assurance and quality control were carried out with parallel determination experiments, blank tests and recovery tests. Blank tests were accompanied in every batch of samples processing. The standard curve was drawn when the correlation coefficient higher than 0.999 for all samples detection. The detection results were reliable when relative deviations of parallel samples analysis below 10%, and the recovery rate ranged from 85% to 110%.

### Bio-concentration factor and correlation analysis

Bio-concentration factor (BCF) was applied in evaluate the accumulate ability of the aquatic organism intake trace elements from aquatic environment, and was established by the United States Environmental Protection Agency [65]. BCF value more than 1 means aquatic organism probably has accumulated trace elements from aquatic environment but not significant unless the BCF value exceed 100. The calculation equation is listed as following:

$$BCF=(C_{F}/C_{W})\times 10^{-3}$$

Where C_F_ is the trace element concentration in fish tissues (mg/kg); C_W_ is the trace element concentration in surface water of Honghu Lake (μg /L).

Correlation matrix can reveal the viable relations among heavy metals, which can provide essential information for source apportionment and pollution pathway analysis. Bivariate procedure is used for the parametric and nonparametric correlation analysis between two or more variables, and the multiple variables calculation results will give out the correlation analysis for each two variables. Pearson correlation matrix is wildly used in bivariate normal distribution data analysis, and Kendall rank correlation coefficient or Spearman rank correlation coefficient is for the other. This article selected Kolmogorov-Smirnov test (K-S test) to analyze whether the data normal distributed.

### Health risk assessment model

Target hazard quotient (abbreviated as THQ) was a kind of assessment method to evaluate the possible non-carcinogenic health risks due to chemical pollutants intake, and was established by the United States Environmental Protection Agency [52]. This method assumes the intake dose is equal to the absorb dose, and cooking has no effect on the pollutants [66]. THQ value less than 1 means there will be no obvious risk in consuming the study fish sample, in another words, the exposure level of the study element is less than the reference dose. The calculation equation is listed as following:

$$THQ=[({\text{E}}_{\text{F}}\times {\text{E}}_{\text{D}}\times {\text{F}}_{\text{IR}}\times \text{C})/({\text{R}}_{\text{FD}}\times {\text{W}}_{\text{AB}}\times {\text{T}}_{\text{A}})]\times 10^{-3}$$

Where E_F_ is the population exposure frequency (350 d/a); E_D_ is the exposure time (30 a); F_IR_ is the food consumption rate (54.33 g/d); C is heavy metal concentration in food (mg/kg); R_FD_ is reference oral dose (mg/kg/d); W_AB_ is the population average weight (61.6 kg); T_A_ is non-carcinogenic average exposure time (365 d/a × E_D_) [67].

Carcinogenic risk (abbreviated as CR) was a kind of assessment method to evaluate the possible carcinogenic health risks due to chemical pollutants intake like As and Pb, and was also established by the United States Environmental Protection Agency [52]. Acceptable carcinogens risk level range was from 10^–4^ (risk of developing cancer over a human lifetime is 1 in 10,000) to 10^–6^ (risk of developing cancer over a human lifetime is 1 in 1,000,000). The calculation equation is listed as following:

$$CR=[({\text{E}}_{\text{F}}\times {\text{E}}_{\text{D}}\times {\text{F}}_{\text{IR}}\times \text{C}\times \text{CSFO})/({\text{W}}_{\text{AB}}\times {\text{T}}_{\text{A}})]\times 10^{-3}$$

Where E_F_ is the population exposure frequency (350 d/a); E_D_ is the exposure time (30 a); F_IR_ is the food consumption rate (54.33 g/d); C is the heavy metal concentration in food (mg/kg); CSFO is the oral carcinogenic slope factor from the Integrated Risk Information System database [68] (mg/kg/d)^−1^; W_AB_ is the population average weight (61.6 kg); T_A_ is the non-carcinogenic average exposure time (365 d/a × E_D_).

Estimated weekly intake (abbreviated as EWI) was employed to calculate the weekly heavy metal intake from food, and was established by the World Health Organization [69] and the United Nations Food and Agriculture Organization [70]. Provisional tolerable weekly intake (abbreviated as PTWI) represents the tentative allowed heavy metal weekly intake. The estimated weekly intake value lower than the provisional tolerable weekly intake value means there will be no obvious risk in consuming the study fish sample. The calculation equation is listed as following:

$$EWI=(F_{IR}\times C\times 7)/W_{AB}$$

Where F_IR_ is the food consumption rate (54.33 g/d); C is the heavy metal concentration in food (mg/kg); W_AB_ is the population average weight (61.6 kg).

## CONCLUSIONS

This study provided valuable information of As, Cd, Cr, Cu, Pb, Zn distribution in different tissues (bladder, gill, intestine, liver, muscle, scale and skin) of various fish (bighead carp, crucian carp, grass carp, mandarin fish, small crucian carp and yellow catfish) captured from Honghu Lake. In general, the heavy metals and metalloid concentrations in muscle of all fish were within the corresponding standards, except the Cr concentration in muscle of crucian carp. There was no significant health risk for the local people through consuming fish muscle and mixed fish tissues, based on the calculation results of THQ, CR, and EWI. For local residents, grass carp was recommended as the daily consuming fish species, which had relatively lower toxic metals accumulation abilities, and followed by yellow catfish and mandarin fish. Fish intestine, liver and bladder were recommended not be added in diet ingredients due to their high accumulation abilities in toxic elements. Consuming less than 1,316 g/d wild fish muscle or 525 g/d mixed wild fish tissues for an individual could basically assure local inhabitants' health. For local government, Pb and Cr were regarded as relatively major contributors for non-carcinogenic risks and were recommended as key factors for aquatic environment regular monitoring in Honghu Lake. Crucian, which had relatively higher bioaccumulation ability in Pb and Cr, was recommended as a sensitive bioindicator in water quality monitoring.

## SUPPLEMENTARY MATERIALS FIGURE AND TABLES



## References

[1] Uluturhan E, Kucuksezgin F (2007). Heavy metal contaminants in Red Pandora (Pagellus erythrinus) tissues from the Eastern Aegean Sea, Turkey. Water Res.

[2] Liang J, Feng CT, Zeng GM, Gao X, Zhong MZ, Li XD, Li X, He XY, Fang YL (2017). Spatial distribution and source identification of heavy metals in surface soils in a typical coal mine city, Lianyuan, China. Environ Pollut.

[3] Liang J, Li XM, Yu ZG, Zeng GM, Luo Y, Jiang L, Yang Z, Qian Y, Wu HP (2017). Amorphous MnO2 modified biochar derived from aerobically composted swine manure for adsorption of Pb(II) and Cd(II). Acs Sustain Chem Eng.

[4] Hua SS, Liang J, Zeng GM, Xu M, Zhang C, Yuan YJ, Li XD, Li P, Liu JY, Huang L (2015). How to manage future groundwater resource of China under climate change and urbanization: An optimal stage investment design from modern portfolio theory. Water Res.

[5] Li F, Huang JH, Zeng GM, Yuan XZ, Li XD, Liang J, Wang XY, Tang XJ, Bai B (2013). Spatial risk assessment and sources identification of heavy metals in surface sediments from the Dongting Lake, Middle China. J Geochem Explor.

[6] Tang WW, Zeng GM, Gong JL, Liu Y, Wang XY, Liu YY, Liu ZF, Chen L, Zhang XR, Tu DZ (2012). Simultaneous adsorption of atrazine and Cu (II) from wastewater by magnetic multi-walled carbon nanotube. Chem Eng J.

[7] Tang WW, Zeng GM, Gong JL, Liang J, Xu P, Zhang C, Huang BB (2014). Impact of humic/fulvic acid on the removal of heavy metals from aqueous solutions using nanomaterials: A review. Sci Total Environ.

[8] Tang WW, Kovalsky P, He D, Waite D (2015). Fluoride and nitrate removal from brackish groundwaters by batch-mode capacitive deionization. Water Res.

[9] Li F, Qiu ZZ, Zhang JD, Liu WC, Liu CY, Zeng GM (2017). Investigation, pollution mapping and simulative leakage health risk assessment for heavy metals and metalloids in groundwater from a typical brownfield, Middle China. Int J Environ Res Pub Health.

[10] Huang JH, Li F, Zeng GM, Liu WC, Huang XL, Xiao ZH, Wu HP, Gu YL, Li X, He XX, He Y (2016). Integrating hierarchical bioavailability and population distribution into potential eco-risk assessment of heavy metals in road dust: A case study in Xiandao District, Changsha city, China. Sci Total Environ.

[11] Ahmed MK, Baki MA, Islam MS, Kundu GK, Habibullah-Al-Mamun M, Sarkar SK, Hossain MM (2015). Human health risk assessment of heavy metals in tropical fish and shellfish collected from the river Buriganga, Bangladesh. Environ Sci Pollut Res.

[12] Leung HM, Leung AOW, Wang HS, Ma KK, Liang Y, Ho KC, Cheung KC, Tohidi F, Yung KKL (2014). Assessment of heavy metals/metalloid (As, Pb, Cd, Ni, Zn, Cr, Cu, Mn) concentrations in edible fish species tissue in the Pearl River Delta (PRD), China. Mar Pollut Bull.

[13] Jia YY, Kong Q, Yang ZG, Wang L (2016). Accumulation behavior and risk assessment of heavy metals and arsenic in tissues of white bream (Parabramis pekinensis) from the Xiang River, southern China. Environ Sci Pollut R.

[14] Yi YJ, Yang ZF, Zhang SH (2011). Ecological risk assessment of heavy metals in sediment and human health risk assessment of heavy metals in fish in the middle and lower reaches of the Yangtze River basin. Environ Pollut.

[15] Li F, Zhang JD, Jiang W, Liu CY, Zhang ZM, Zhang CD, Zeng GM (2017). Spatial health risk assessment and hierarchical risk management for mercury in soils from a typical contaminated site, China. Environ Geochem Hlth.

[16] Agency for Toxic Substances and Disease Registry (2003). Toxicological Profile for Arsenic.

[17] Castro-Gonzalez MI, Mendez-Armenta M (2008). Heavy metals: implications associated to fish consumption. Environ Toxicol Phar.

[18] Garcia-Leston J, Mendez J, Pasaro E, Laffon B (2010). Genotoxic effects of lead: an updated review. Environ Int.

[19] United States Food and Drug Administration (1993). Guidance document for arsenic in shellfish.

[20] Flemming CA, Trevors JT (1989). Copper toxicity and chemistry in the environment: a review. Water Air Soil Pollut.

[21] Schmitt CJ, Brumbaugh WG (1990). National contaminant biomonitoring program: concentrations of arsenic, cadmium, copper, lead, mercury, selenium, and zinc in U.S. freshwater fish. Arch Environ Con Tox.

[22] Makokha VA, Qi YL, Shen Y, Wang J (2016). Concentrations, distribution, and ecological risk assessment of heavy metals in the east Dongting and Honghu Lake, China. Expos Health.

[23] Li F, Qiu ZZ, Zhang JD, Liu CY, Cai Y, Xiao MS (2017). Spatial distribution and fuzzy health risk assessment of trace elements in surface water from Honghu Lake. Int J Environ Res Pub Health.

[24] Ban X, Wu QZ, Pan BZ, Du Y, Feng Q (2014). Application of composite water quality identification index on the water quality evaluation in spatial and temporal variations: a case study in Honghu Lake, China. Environ Monit Assess.

[25] Li T, Lei WX, Bo WC, Fang F (2009). Effect of enclosure culture on water quality: a case study in Lake Honghu, Hubei province, China. 2009 International Conference on Environmental Science and Information Application Technology.

[26] Hu Y, Qi SH, Wu CX, Ke YP, Chen J, Chen W, Gong XY (2012). Preliminary assessment of heavy metal contamination in surface water and sediments from Honghu Lake, East Central China. Front Earth Sci.

[27] Honghu City Government China (2016). The statistics of primary products inHonghu area by the local government. http://www.honghu.gov.cn/z/mlhh/hhgl/zycw/2015-07-10/66.html.

[28] Honghu City Government China (2016). Statistical Communique on national economic and social development of Honghu Municipality in 2016.

[29] Jiang HF, Qin DL, Chen ZX, Tang SZ, Bai SY, Mou ZB (2016). Heavy metal levels in fish from Heilongjiang River and potential health risk assessment. B Environ Contam Tox.

[30] Yipel M, Turk E, Tekeli IO, Oguz H (2016). Heavy metal levels in farmed and wild fish of Aegean Sea and assessment of potential risks to human health. Kafkas Üniversitesi Veteriner Fakültesi Dergisi.

[31] Cheng Z, Lam CL, Mo WY, Nie XP, Choi WM, Man YB, Wong MH (2016). Food wastes as fish feeds for polyculture of low-trophic level fish: bioaccumulation and health risk assessments of heavy metals in the cultured fish. Environ Sci Pollut R.

[32] Hu Y, Zhou CS, Hu LH, Pan QC, Jiang QQ, Wu Y, Wang YH, Zheng YN, Dai Y (2015). [Comparative analysis of the nutritional composition in the muscles and skins of Anguilla Japonica cultured in the seawater and freshwater]. [Article in Chinese]. Acta Hydrobiol Sin.

[33] Su S (2001). Fish is also a kind of delicacies. Dietary guidelines.

[34] Phoenix News (2017). The rank list of the most popular food in Hubei. http://share.iclient.ifeng.com/news/shareNews?forward=1=118727110#backhead.

[35] Adhikari S, Ghosh L, Giri BS, Ayyappn S (2009). Distribution of metals in the food web of fishponds of Kolleru Lake, India. Ecotox Environ Safe.

[36] Avigliano E, Lozano C, Pla RR, Volpedo AV (2016). Toxic element determination in fish from Parana River Delta (Argentina) by neutron activation analysis: tissue distribution and accumulation and health risk assessment by direct consumption. J Food Compos Anal.

[37] Wei YH, Zhang JY, Zhang DW, Tu TH, Luo LG (2014). Metal concentrations in various fish organs of different fish species from Poyang Lake, China. Ecotox Environ Safe.

[38] Ministry of Health, PRC (2012). National food safety standard: Contaminants Limitation in Foods. GB2762–2012.

[39] Ministry of Agriculture, PRC (2006). National Agriculture standard: Toxic and hazardous substances Limitation in edible aquatic products.

[40] Chi QQ, Zhu GW, Alan L (2007). Bioaccumulation of heavy metals in fish from Taihu Lake, China. J Environ Sci.

[41] Rahman MS, Molla AH, Saha N, Rahman A (2012). Study on heavy metals levels and its risk assessment in some edible fish from Bangshi River, Savar, Dhaka, Bangladesh. Food Chem.

[42] De TK, De M, Das S, Ray R, Ghosh PB (2010). Level of heavy metals in some edible marine fish of mangrove dominated tropical estuarine areas of Hooghly River, north east coast of Bay of Bengal, India. B Environ Contam Tox.

[43] Pintaeva ETS, Bazarsadueva SV, Radnaeva LD, Pertov EA, Smirnova OG (2011). Content and character of metal accumulation in fish of the Kichera River (a tributary of Lake of Baikal). Contemp Probl Ecol.

[44] Varol M, Kaya GK, Alp A (2017). Heavy metal and arsenic concentrations in rainbow trout (Oncorhynchus mykiss) farmed in a dam reservoir on the Firat (Euphrates) River: Risk-based consumption advisories. Sci Total Environ.

[45] Alamdar A, Eqani SA, Hanif N, Ali SM, Fasola M, Bokhari H, Katsoyiannis LA, Shen HQ (2017). Human exposure to trace metals and arsenic via consumption of fish from river Chenab, Pakistan and associated health risks. Chemosphere.

[46] Alam L, Mokhtar MB, Alam MM, Bari MA, Kathijotes N, Ta GC, Lee KE (2015). Assessment of environmental and human health risk for contamination of heavy metal in tilapia fish collected from Langat Basin, Malaysia. Asian J Water Environ Pollut.

[47] Santerre CR, Bush PB, Xu DH, Lewis GW, Davis JT, Grodner RM, Ingram R, Wei CI, Hinshaw JM (2001). Metal residues in farm-raised channel catfish, rainbow trout, and red swamp crayfish from the Southern U.S. J Food Sci.

[48] Fallah AA, Saei-Dehkordi SS, Nematollahi A, Jafari T (2011). Comparative study of heavy metal and trace element accumulation in edible tissues of farmed and wild rainbow trout (Oncorhynchus mykiss) using ICP-OES technique. Microchem J.

[49] Islam MS, Ahmed MK, Al-Mamun MH, Masunaga S (2015). Assessment of trace metals in fish species of urban rivers in Bangladesh and health implications. Environ Toxicol Phar.

[50] Jayaprakash M, Kumar RS, Giridharan L, Sujitha SB, Sarkar SK, Jonathan MP (2015). Bioaccumulation of metals infish species from water and sediments in macrotidal Ennore creek, Chennai, SE coast of India: A metropolitan city effect. Ecotox Environ Safe.

[51] Jaric I, Visnjic-Jeftic Z, Cvijanovic G, Gacic Z, Jovanovic L, Skoric S, Lenhardt M (2011). Determination of differential heavy metal and trace metal accumulation in liver, gills, intestine and muscle of starlet (Acipenser ruthenus) from the Danube River in Sebia by ICP-OES. Microchem J.

[52] USEPA (1989). Risk assessment guidance for superfund volume 1: human health evaluation manual (part A).

[53] Duan XL, Wang LM, Jiang Y, Zhao XG, Wang BB, Guo J (2013). The fish and shrimps intake rate in different provinces of China. Environmental Protection Department. Exposure Factors Handbook of Chinese Pollution.

[54] Pazi I, Gonul T, Kucuksezgin F, Avaz G, Tolun L, Unluoglu A, Karaaslan Y, Gucver SM, Orhon AK, Siltu E, Olmez G (2017). Potential risk assessment of metals in edible fish species for human consumption from the Eastern Aegean Sea. Mar Pollut Bull.

[55] Honghu City Government China (2013). Nature resources statistics reports for Honghu Lake. http://www.honghu.gov.cn/z/mlhh/hhgl/zrzy/2015-07-10/69.html.

[56] Honghu City Government China (2013). Production resources statistics reports for Honghu Lake. http://www.honghu.gov.cn/z/mlhh/hhgl/zycw/2015-07-10/66.html.

[57] Ministry of Environmental Protection, PRC (2013). Water quality digestion of total metals nitric acid digestion method. HJ 677–2013.

[58] Ministry of Environmental Protection, PRC (2014). Water quality determination of mercury, arsenic, selenium, bismuth and antimony atomic fluorescence spectrometry. HJ.

[59] Ministry of Health, PRC (2010). National food safety standard: Determination of lead in foods. GB 5009.12–2000.

[60] Ministry of Health and Family Planning Commission, PRC (2014). National food safety standard: Determination of cadmium in foods. GB 5009.15–2014.

[61] Ministry of Health and Family Planning Commission, PRC (2014). National food safety standard: Determination of chromium in foods. GB 5009.123–2014.

[62] Ministry of Health and Family Planning Commission, PRC (2014). National food safety standard: Determination of total arsenic and inorganic arsenic in foods. GB 5009.11–2014.

[63] Ministry of National Standardization Management Committee, PRC (2003). National food safety standard: Determination of copper in foods. GB/T 5009.13–2003.

[64] Ministry of National Standardization Management Committee, PRC (2003). National food safety standard: Determination of Zinc in foods. GB/T 5009.14–2003.

[65] USEPA (1991). Technical Support Document For Water Quality-based Toxics Control.

[66] Cooper CB, Doyle ME, Kipp K (1991). Risk of consumption of contaminated seafood, the Quincy Bay Case Study. Environ Health Persp.

[67] Zeng XX, Liu YG, You SH, Zeng GM, Tan XF, Hu XJ, Hu X, Huang L, Li F (2015). Spatial distribution, health risk assessment and statistical source identification of the trace elements in surface water from the Xiangjiang River, China. Environ Sci Pollut R.

[68] USEPA (2010). Risk-based concentration table.

[69] World Health Organization (2004). Guidelines for Drinking Water Quality, 3rd ed.

[70] FAO (2006). Arsenic Contamination of Irrigation Water, Soil and Crops in Bangladesh: Risk Implications for Sustainable Agriculture.

