# Supplementary material for: Heavy metals and metalloid distribution in different organs and health risk assessment for edible tissues of fish captured from Honghu Lake
Source: Oncotarget. 2017 Oct 13;8(60):101672–85. doi: 10.18632/oncotarget.21901 (PMC5731905; doi:10.18632/oncotarget.21901)
Supplement: Supplementary file 1 [file oncotarget-08-101672-s001.pdf]

Heavy metals and metalloid distribution in different organs and health risk assessment for edible tissues of fish captured from Honghu Lake

SUPPLEMENTARY MATERIALS

Supplementary Table 1: The Pearson correlation analysis between different trace elements in fish muscle

	As	Cd	Cr	Cu	Pb	Zn
As	1.000					
Cd	-0.267	1.000				
Cr	-0.382	0.418	1.000			
Cu	0.655	-0.572	-0.319	1.000		
Pb	0.356	0.448	0.008	0.423	1.000	
Zn	0.144	-0.526	-0.079	0.834*	0.275	1.000

* Correlation is significant at $P < 0.05$

Supplementary Table 2: The Pearson correlation analysis between different fish organs in the same trace element

	Bladder	Gill	Intestine	Liver	Muscle	Scale	Skin
**As**							
Bladder	1.000						
Gill	0.684	1.000					
Intestine	0.489	0.783*	1.000				
Liver	0.108	-0.260	-0.204	1.000			
Muscle	-	-	-	-	1.000		
Scale	0.111	0.434	0.882**	-0.143	-	1.000	
Skin	-0.364	-0.041	-0.280	-0.204	-	-0.0204	1.000
**Cd**							
Bladder	1.000						
Gill	0.496	1.000					
Intestine	-0.207	-0.391	1.000				
Liver	-0.069	-0.068	0.129	1.000			
Muscle	-0.534	-0.094	-0.113	0.367	1.000		
Scale	0.306	0.453	-0.203	0.498	0.457	1.000	
Skin	-0.569	-0.054	-0.144	-0.427	0.503	-0.007	1.000
**Cr**							
Bladder	1.000						
Gill	0.255	1.000					
Intestine	-0.474	-0.329	1.000				
Liver	0.484	-0.310	0.108	1.000			
Muscle	0.095	0.761*	-0.158	-0.426	1.000		
Scale	-0.382	-0.129	-0.034	-0.351	-0.328	1.000	
Skin	-0.382	0.488	0.298	-0.442	-0.283	0.399	1.000
**Cu**							
Bladder	1.000						
Gill	0.877**	1.000					
Intestine	0.494	0.267	1.000				
Liver	-0.289	0.168	-0.495	1.000			
Muscle	0.299	0.010	0.174	-0.684	1.000		
Scale	-0.461	-0.371	-0.295	0.352	-0.458	1.000	
Skin	0.346	0.454	0.116	-0.001	0.162	-0.030	1.000
**Pb**							
Bladder	1.000						
Gill	0.184	1.000					
Intestine	-0.296	-0.076	1.000				
Liver	0.184	-0.232	-0.475	1.000			
Muscle	-0.281	0.475	0.474	-0.037	1.000		
Scale	0.119	-0.355	-0.611	0.500	-0.430	1.000	
Skin	-0.245	0.506	0.233	-0.868**	0.189	-0.548	1.000
**Zn**							
Bladder	1.000						
Gill	-0.143	1.000					
Intestine	0.548	0.381	1.000				
Liver	0.425	0.554	0.889**	1.000			
Muscle	0.316	-0.058	0.709*	0.430	1.000		
Scale	0.448	0.402	0.308	0.154	0.220	1.000	
Skin	0.238	0.619	0.619	0.477	0.561	0.674	1.000

*Correlation is significant at $P < 0.05$
**Correlation is significant at $P < 0.01$

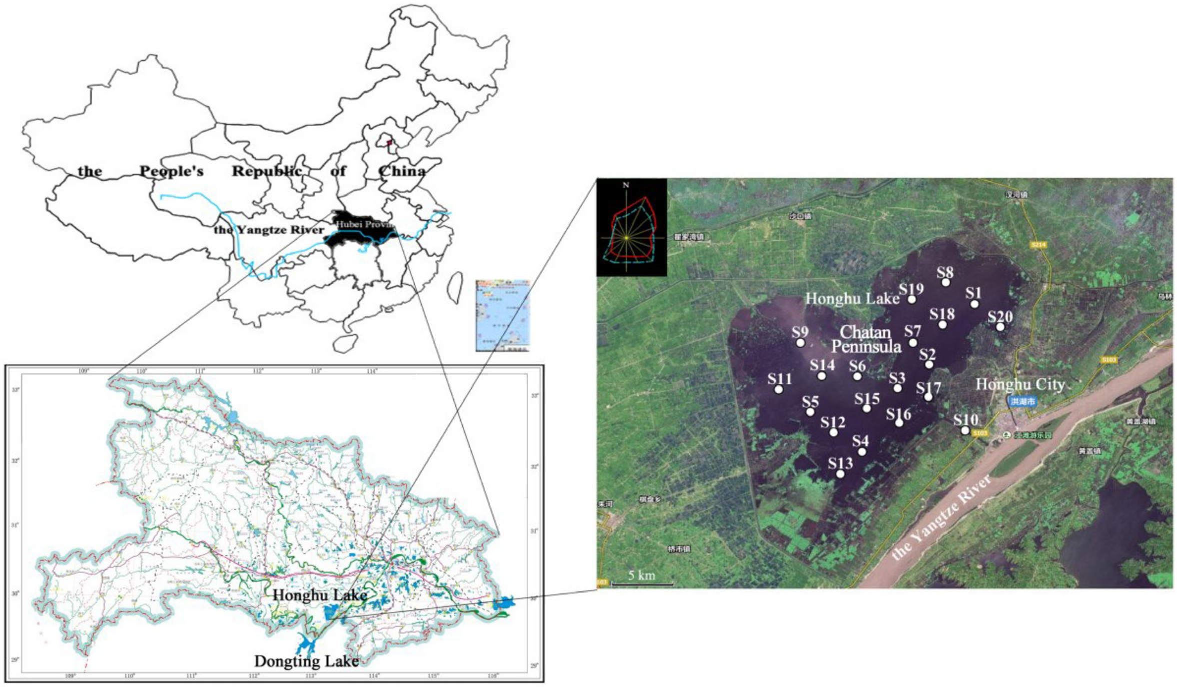

Supplementary Figure 1: Map of surface water sampling sites in Honghu Lake [23].
